# The 2023 Impact of Inflammatory Bowel Disease in Canada: Special Populations—IBD in Seniors

**DOI:** 10.1093/jcag/gwad013

**Published:** 2023-09-05

**Authors:** Seth R Shaffer, M Ellen Kuenzig, Joseph W Windsor, Alain Bitton, Jennifer L Jones, Kate Lee, Sanjay K Murthy, Laura E Targownik, Juan-Nicolás Peña-Sánchez, Noelle Rohatinsky, Sara Ghandeharian, Parul Tandon, Joëlle St-Pierre, Navneet Natt, Tal Davis, Jake Weinstein, James H B Im, Eric I Benchimol, Gilaad G Kaplan, Quinn Goddard, Julia Gorospe, Maxime Bergevin, Ken Silver, Dawna Bowles, Margaret Stewart, Marsha Pearlstein, Elizabeth H Dawson, Charles N Bernstein

**Affiliations:** Department of Internal Medicine, Max Rady College of Medicine, Rady Faculty of Health Sciences, University of Manitoba, Winnipeg, Manitoba, Canada; University of Manitoba IBD Clinical and Research Centre, Winnipeg, Manitoba, Canada; SickKids Inflammatory Bowel Disease Centre, Division of Gastroenterology, Hepatology, and Nutrition, The Hospital for Sick Children, Toronto, Ontario, Canada; Child Health Evaluative Sciences, SickKids Research Institute, The Hospital for Sick Children, Toronto, Ontario, Canada; Departments of Medicine and Community Health Sciences, University of Calgary, Calgary, Alberta, Canada; Division of Gastroenterology and Hepatology, McGill University Health Centre IBD Centre, McGill University, Montréal, Quebec, Canada; Departments of Medicine, Clinical Health, and Epidemiology, Dalhousie University, Halifax, Nova Scotia, Canada; Crohn’s and Colitis Canada, Toronto, Ontario, Canada; Department of Medicine, University of Ottawa, Ottawa, Ontario, Canada; The Ottawa Hospital IBD Centre, Ottawa, Ontario, Canada; Division of Gastroenterology and Hepatology, Mount Sinai Hospital, University of Toronto, Toronto, Ontario, Canada; Department of Community Health and Epidemiology, University of Saskatchewan, Saskatoon, Saskatchewan, Canada; College of Nursing, University of Saskatchewan, Saskatoon, Saskatchewan, Canada; Crohn’s and Colitis Canada, Toronto, Ontario, Canada; Division of Gastroenterology and Hepatology, University Health Network, University of Toronto, Toronto, Ontario, Canada; Departments of Medicine and Community Health Sciences, University of Calgary, Calgary, Alberta, Canada; Northern Ontario School of Medicine University, Sudbury, Ontario, Canada; SickKids Inflammatory Bowel Disease Centre, Division of Gastroenterology, Hepatology, and Nutrition, The Hospital for Sick Children, Toronto, Ontario, Canada; Child Health Evaluative Sciences, SickKids Research Institute, The Hospital for Sick Children, Toronto, Ontario, Canada; SickKids Inflammatory Bowel Disease Centre, Division of Gastroenterology, Hepatology, and Nutrition, The Hospital for Sick Children, Toronto, Ontario, Canada; Child Health Evaluative Sciences, SickKids Research Institute, The Hospital for Sick Children, Toronto, Ontario, Canada; SickKids Inflammatory Bowel Disease Centre, Division of Gastroenterology, Hepatology, and Nutrition, The Hospital for Sick Children, Toronto, Ontario, Canada; Child Health Evaluative Sciences, SickKids Research Institute, The Hospital for Sick Children, Toronto, Ontario, Canada; SickKids Inflammatory Bowel Disease Centre, Division of Gastroenterology, Hepatology, and Nutrition, The Hospital for Sick Children, Toronto, Ontario, Canada; Child Health Evaluative Sciences, SickKids Research Institute, The Hospital for Sick Children, Toronto, Ontario, Canada; ICES, Toronto, Ontario, Canada; Department of Paediatrics, Temerty Faculty of Medicine, University of Toronto, Toronto, Ontario, Canada; Institute of Health Policy, Management, and Evaluation, Dalla Lana School of Public Health, University of Toronto, Toronto, Ontario, Canada; Departments of Medicine and Community Health Sciences, University of Calgary, Calgary, Alberta, Canada; Departments of Medicine and Community Health Sciences, University of Calgary, Calgary, Alberta, Canada; Departments of Medicine and Community Health Sciences, University of Calgary, Calgary, Alberta, Canada; École de kinésiologie et des sciences de l’activité physique, Faculté de médecine, Université́ de Montréal, Montreal, Quebec, Canada; Centre de recherche de l’Institut universitaire de gériatrie de Montréal, Montreal, Quebec, Canada; Crohn’s and Colitis Canada, Toronto, Ontario, Canada; Crohn’s and Colitis Canada, Toronto, Ontario, Canada; Crohn’s and Colitis Canada, Toronto, Ontario, Canada; Crohn’s and Colitis Canada, Toronto, Ontario, Canada; Crohn’s and Colitis Canada, Toronto, Ontario, Canada; Department of Internal Medicine, Max Rady College of Medicine, Rady Faculty of Health Sciences, University of Manitoba, Winnipeg, Manitoba, Canada; University of Manitoba IBD Clinical and Research Centre, Winnipeg, Manitoba, Canada

**Keywords:** Crohn’s disease, Ulcerative colitis, Elderly, Comorbidity, Risks

## Abstract

Approximately one out of every 88 seniors has inflammatory bowel disease (IBD), and this is expected to increase in the future. They are more likely to have left-sided disease in ulcerative colitis, and isolated colonic disease in Crohn’s disease; perianal disease is less common. Other common diagnoses in the elderly must also be considered when they initially present to a healthcare provider. Treatment of the elderly is similar to younger persons with IBD, though considerations of the increased risk of infections and malignancy must be considered when using immune modulating drugs. Whether anti-TNF therapies increase the risk of infections is not definitive, though newer biologics, including vedolizumab and ustekinumab, are thought to be safer with lower risk of adverse events. Polypharmacy and frailty are other considerations in the elderly when choosing a treatment, as frailty is associated with worse outcomes. Costs for IBD-related hospitalizations are higher in the elderly compared with younger persons. When elderly persons with IBD are cared for by a gastroenterologist, their outcomes tend to be better. However, as elderly persons with IBD continue to age, they may not have access to the same care as younger people with IBD due to deficiencies in their ability to use or access technology.

**Key Points**

1. In 2023, one out of every 88 elderly persons has IBD, with the prevalence expected to increase in coming years.
2. Although the evidence is conflicting, anti-TNF use is potentially associated with an increased risk of infection, including pneumonia.
3. Newer biologic therapies, including vedolizumab and ustekinumab, are promising therapies for the elderly with fewer infectious adverse events.
4. The complex healthcare needs of an aging IBD population are placing additional stress on specialist gastroenterology clinics to adequately care for these individuals.
5. Increasing comorbidities and frailty evident in the elderly may impact their ability to handle the symptoms that IBD imparts, and in turn, may impact possible treatment options.
6. The elderly are more likely to be using multiple prescription drugs and hence there is a greater likelihood of drug interactions and potential adverse drug-related side effects.
7. The complex healthcare needs of this population require multidisciplinary healthcare teams in order to adequately care for the sequelae of aging.
8. It is particularly challenging for the elderly to access care due to mobility restrictions, logistical challenges, or lack of advocacy support. Therefore, virtual access (either by video or phone) should be offered as an option.
9. Vaccines, including the pneumococcus and COVID-19 vaccines among others, can provide protection against infections in the elderly with IBD.

## Summary of Crohn’s and Colitis Canada’s 2018 Impact of IBD: Special Populations—IBD in Seniors

In 2018, one out of every 160 seniors had inflammatory bowel disease (IBD); 15% of all people with IBD are diagnosed over the age of 65. Seniors diagnosed with Crohn’s disease are more likely to have isolated colonic disease without fistulizations, compared with those diagnosed at a younger age. The use of thiopurines was associated with a higher risk of lymphoma, when compared to those under the age of 50 exposed to thiopurines. The use of anti-TNF therapy was lower for both Crohn’s disease and ulcerative colitis, compared with younger people with IBD. Persons between the ages of 65–79 with IBD had higher healthcare costs when compared with age-matched controls.

## INTRODUCTION

One out of every 88 seniors is diagnosed with inflammatory bowel disease (IBD), and the prevalence is only increasing. Seniors diagnosed with this chronic disease face distinct challenges in treatment due to co-existing co-morbidities, polypharmacy, frailty and an increased risk of infections and malignancies. Further, their presentation, presenting phenotype, and natural history differ from adults with IBD. Vaccines are an important tool to prevent or decrease the severity of infections in seniors with IBD, including vaccines against SARS-CoV-2. The treatment and management of seniors with IBD are quite challenging due to these many factors which makes it an important healthcare concern in Canada as the population continues to age.

## EPIDEMIOLOGY

While the majority of individuals with IBD are diagnosed as teenagers or young adults, as many as 10% to 15% are diagnosed over the age of 60 (1). In 2023, we estimate one out of every 88 elderly persons in Canada has IBD (1.14% of seniors), with prevalence increasing by 2.76% (95% CI: 2.73, 2.79) per year due to a combination of new diagnoses and the aging of individuals diagnosed with IBD earlier in life (2). The incidence rate of IBD among seniors is 28.7 (95% CI: 24.4, 33.0) per 100,00 person-years and is not changing over time (average annual percentage change: 0.66; 95% CI: −0.55, 1.52) (2). A retrospective study of people with ulcerative colitis in South Korea revealed that ulcerative colitis-related and all-cause mortality was higher in the elderly-onset group compared to the nonelderly group (3). Diagnosing IBD in the elderly can be challenging as other conditions mimicking IBD must be ruled out (4); these include colorectal cancer, ischemic colitis, segmental colitis with associated diverticulosis, radiation enteritis or colitis, and microscopic colitis (4). Elderly persons with IBD are at increased risk of mortality, based on data from a Swedish National Register Study (5).

## PHENOTYPE

Elderly people with Crohn’s disease are most likely to have isolated colonic disease; penetrating or perianal disease is rare (1,6,7). The rate of progression to stricturing or penetrating disease is either similar, or lower, than in adults with Crohn’s disease (6). In those with ulcerative colitis, left-sided disease is the most common presentation (6).

## SURGERY

In a systematic review and meta-analysis, the cumulative five-year risk of surgery in the people with Crohn’s disease over the age of 60 is 22.6%. This same study reported that the cumulative five-year risk of surgery in elderly persons with ulcerative colitis was 7.8% (6). The five-year risk of surgery was similar in the elderly and adults with IBD. Age >60 years was found to be associated with a decreased risk of using prophylactic therapies after an ileo-cecal resection (odds ratio [OR]: 0.20; 95% CI: 0.05, 0.76) (8). In a study of 148 persons hospitalized with moderate-to-severe ulcerative colitis, persons >60 year, mostly on intravenous corticosteroids, experienced more treatment failures compared with those younger than 60 years old, leading to more colectomies (28.4% vs. 12.2%) (9).

## DISEASE-RELATED COMPLICATIONS

In one study by Rozich et al., there was no difference in disease-related complications (IBD-related surgery, hospitalization, treatment escalation, clinical flare, or disease complication) in elderly persons (>60 years) with either incident or prevalent IBD compared with adults aged 18 to 59 with IBD (adjusted hazard ratio [aHR]: 0.85; 95% CI: 0.58, 1.25) (10). Elderly individuals were also less likely to experience treatment-related complications (including serious infection, malignancy, or death) (aHR: 0.58; 95% CI: 0.39, 0.87). A retrospective study from 13 hospitals in Hong Kong revealed that elderly-onset IBD had an increased risk of cytomegalovirus colitis (OR: 3.07; 95% CI: 1.92, 4.89), herpes zoster infection (OR: 2.42; 95% CI: 1.22, 4.80), all-cancer development (OR: 2.97; 95% CI: 1.84, 4.79), and IBD-related hospitalizations (OR: 1.14; 95% CI: 1.09, 1.20), compared with people with adult-onset IBD (7). In a large US cohort study using health administrative data from Veterans Affairs, the incidence of herpes zoster infection was higher in persons with IBD (treated with 5-ASA only) when compared to those without IBD (aHR: 1.72; 95% CI: 1.51, 1.96), and within IBD, age was a risk factor for developing herpes zoster (11). A retrospective study assessing a nationwide database in the US reported that elderly persons (older than 64 years) with IBD when compared with middle-aged (40 to 64 years old) and younger individuals (younger than 40) were more likely to be hospitalized due to serious infections (14.6% vs. 10.6% and 8.4%), and cardiovascular complications (9.9% vs. 4.3% and 0.8%), respectively (12). In a market-scan database study from the US, 63,759 persons with IBD were identified, and infections were higher in the elderly group (65 years old or older) (incidence rate [IR]: 16.95 per 100 person-years vs. 10.49 per 100 person-years), with pneumonia (39.8%), sepsis (13.2%), and candidiasis (12.9%) being the most common infections (13). Individuals with IBD-related hospitalizations who are 65 or older have an increased risk of mortality (OR: 3.91; 95% CI: 2.50, 6.11), compared to those with IBD who are 19 to 64 years old, after controlling for comorbidities and complications, and this risk is even more pronounced when compared with those between 19 and 35 years old (OR: 17.42; 95% CI: 8.92, 33.99) (14).

## TREATMENT

The medical management of elderly persons with IBD is similar to younger people with IBD. However, age, polypharmacy, comorbidities, and risk of frailty complicate disease management. Therapeutic efficacy is thought to be similar between the elderly and younger persons with IBD, but one must balance the increased of risk of infection, potential drug interactions, and heightened risk of malignancy (15).

Mesalamine has been demonstrated to be an effective treatment for the induction and maintenance of remission in mild-to-moderate ulcerative colitis, though the evidence is lacking for its use in Crohn’s disease. Despite this, mesalamine is used in more than two-thirds of elderly persons with both Crohn’s disease and ulcerative colitis (16,17). Thiopurine use is a convenient oral medication for maintaining remission in both Crohn’s disease and ulcerative colitis, with one study indicating approximately 20% of the cohort being exposed to a thiopurine within five years of IBD diagnosis (18).

In a systematic review and meta-analysis comparing elderly-onset persons with IBD (>60 years) with adult-onset persons with IBD, the use of corticosteroids was shown to be similar between these two groups, though the use of immunomodulators and biologics was lower in the elderly group for both Crohn’s disease and ulcerative colitis (6).

## EFFICACY/EFFECTIVENESS

Data on the effectiveness of anti-TNF therapy in the elderly are conflicting (Table 1). Compared to younger people with IBD, older persons had lower persistence on their anti-TNFs, were less likely to achieve corticosteroid-free remission at 12 months (31% vs. 67%, *P* < 0.001), and were more likely to experience anti-TNF failure (19,20). Adverse events, rather than lack of effectiveness, were more often the reason for anti-TNF cessation in older adults; thiopurine use was associated with a lower risk of treatment failure (20). Conversely, a pooled analysis of data from randomized trials assessing anti-TNF use in ulcerative colitis reported no significant differences in the efficacy of anti-TNF in both inducing and maintaining remission between the older cohort (≥60 years) and younger cohort (<60 years) (OR: 1.05; 95% CI: 0.33, 3.39) (21). Infliximab exposure, including levels and antibodies, is thought to be the same in elderly persons, hence, similar trough concentrations can be used to guide therapy (22). A post hoc analysis of the Randomized Evaluation of an Algorithm for Crohn’s Treatment (REACT) study, including the subset of participants >60 years with Crohn’s disease, showed no differences in achieving corticosteroid-free clinical remission between early use of combination therapy (anti-TNF agent and thiopurines) and conventional treatment (<60 years, risk ratio [RR]: 1.06; 95% CI: 0.98, 1.15; ≥60 years, RR: 1.09; 95% CI: 0.90, 1.33, *P* = 0.78) (23).

**Table 1.** Effectiveness of inflammatory bowel disease (IBD) therapies between elderly and nonelderly persons with IBD

IBD therapy	Study	Definition of effectiveness	Comparison groups	Type of IBD	Effectiveness
Anti-TNF					
	Porcari et al. (19).	Persistence of therapy at 12 months CS-free remission	≥ 60 years old vs. <60 years old (reference)	IBD	Lower persistence in older cohort: UC (*P* < 0.001) CD (*P* = 0.004) CS-free remission: 31% vs. 67%, *P* < 0.001
	Cheng et al. (21).	Clinical remission	≥ 60 years old vs. <60 years old (reference)	UC	Induction: OR: 0.78; 95% CI: 0.51, 1.19 Maintenance: OR: 0.65; 95% CI: 0.41, 1.06
	De Jong et al. (20).	Discontinuation due to lack of effectiveness (combination of primary and secondary nonresponse)	≥ 60 years old vs. <40 years-old (reference)	IBD	SHR: 1.11; 95% CI: 0.63, 1.95
	Kantasiripitak et al. (22).	Infliximab levels and antibodies	≥ 65 years old vs. <65 years old (reference)	IBD	Infliximab levels at weeks 2, 6, and 14 were similar: *P* = 0.90, *P* = 0.757, *P* = 0.121, respectively Similar rates of antibodies
	Singh et al. (23).	Corticosteroid-free clinical ­remission	≥ 60 years old vs. <60 years old (reference)	CD	CS-free remission: RR: 1.09; 95% CI: 0.90, 1.33
Vedolizumab					
	Ibraheim et al. (24).	Clinical response, clinical remission, and corticosteroid-free remission at 52 weeks	≥ 60 years old	IBD	Clinical response: 52% Clinical remission: 38% CS-free remission: 32%
	Khan et al. (25).	Corticosteroid-free remission, IBD-related hospitalization and IBD-related surgery	≥ 60 years old vs. <60 years old (reference)	IBD	CS-free remission: 40.1% vs. 46.8%, *P* = 0.2374 IBD-related hospitalization: 11.3% vs. 11.2%, *P* = 0.9737 IBD-related surgery: 3.9% vs. 3.9%, *P* = 0.9851
	Cohen et al. (26).	Corticosteroid-free clinical remission and endoscopic remission at 52 weeks	≥ 60 years old vs. ≤40 years (reference)	IBD	CS-free remission: CD: *P* = 0.45 and UC: *P* = 0.54 Endoscopic remission: CD *P* = 0.74 and UC *P* = 0.52
Vedolizumab vs. anti-TNF					
	Pabla et al. (27).	Five-year surgical intervention and IBD-hospitalization free survival, endoscopic remission between vedolizumab and anti-TNF users	≥ 60 years old (anti-TNF users reference group)	IBD	Surgical intervention: *P* = 1.0 IBD-hospitalization: *P* = 0.21 Endoscopic remission: 65.7 vs. 45.2%, *P* = 0.02
	Adar et al. (28).	Clinical remission at 3, 6, and 12 months between vedolizumab and anti-TNF users	≥ 60 years old (anti-TNF users reference group)	IBD	Clinical remission at 3 months in CD: 41% vs. 56%; OR: 2.82, 95% CI: 1.18, 6.76. In UC: 35% vs. 43%; OR: 1.74; 95% CI: 0.74, 4.13 Clinical remission at 6 months: 45% vs. 54%, *P* = 0.23 Clinical remission at 12 months: 54% vs 58%, *P* = 0.63
Ustekinumab					
	Garg et al. (29).	Complete clinical remission, and mucosal healing	≥ 65 years old vs. <65 years old (reference)	CD	Clinical remission: 28.2% vs. 52.6%, *P* = 0.03 Mucosal healing: 26% vs 30%, *P* = 0.74

Vedolizumab, a gut-specific monoclonal antibody, is considered effective in the elderly (24,30), comparable with younger persons (25). In a retrospective study of 144 persons with IBD who were 60 years old or older compared with 140 persons who were 40 years old or younger, there were no differences in clinical or endoscopic response between the two groups (week 52 remission in Crohn’s disease: 40% vs. 35%, *P* = 0.7, week 52 remission in ulcerative colitis: 48% vs. 51%, *P* = 0.84) (26). In a retrospective study of persons over 60 years old comparing 108 persons on vedolizumab with 104 on anti-TNF therapy, there was no difference in surgical intervention or the risk of IBD hospitalization. However, vedolizumab was discontinued less frequently than anti-TNF therapy (25.9% vs. 51.9%, *P* = 0.02). In those who underwent endoscopic evaluation, the vedolizumab cohort had higher endoscopic response (80.0% vs. 59.3%, *P* = 0.02) and endoscopic remission rates (65.7% vs. 45.2%, *P* = 0.02) (27). A retrospective study of persons >60 years treated with either anti-TNF or vedolizumab found that more persons with Crohn’s disease (but not ulcerative colitis) on anti-TNF therapy were in remission at three months, though there was no difference between anti-TNF therapy and vedolizumab in either Crohn’s disease or ulcerative colitis at six and 12 months (28).

Ustekinumab, an antibody directed toward IL-12/23, has similar efficacy in all age groups (31). In a retrospective study of 117 persons with Crohn’s disease, mucosal healing was similar in the elderly cohort (>65 years) compared to those <65 years (26% vs. 30%, *P* = 0.74) (29).

## SAFETY

### Thiopurines

Thiopurine use in persons >65 years is associated with both an increase in nonmelanoma skin cancers (32), and lymphoproliferative disorders (33), when compared to thiopurine users <50 years. Exposure to thiopurines also increases one’s risk of infections (34). A cohort study of 48,752 persons with IBD from Spain, revealed that elderly persons (>60 years) using thiopurines had higher rates of myelotoxicity, digestive intolerance, and hepatotoxicity, and the thiopurines were more likely to be discontinued than in those <60 years (67.2% vs. 63.1%) (35).

A nationwide retrospective study of persons with IBD with a mean age of 63.0, using the Veterans Affairs dataset, revealed that exposure to thiopurines (OR: 0.962; 95% CI: 0.230, 4.027) or anti-TNF therapy (OR: 0.581; 95% CI: 0.174, 1.939) was not associated with an increased risk of contracting COVID-19 (36).

### Biologics

Anti-TNF use in older persons with IBD has been associated with serious infections (aHR: 3.92; 95% CI: 1.185, 12.973), when adjusting for age at diagnosis and number of comorbidities (37). A prospective IBD registry revealed elderly (>60 years) persons on anti-TNF had higher rates of serious adverse events (incidence rate ratio [IRR]: 2.06; 95% CI: 1.42, 3.00), and serious infections (61.2 vs. 12.4 infections per 1000 person-years) compared with a younger cohort (<40 years) (20). A retrospective study of people with IBD using the American Veteran Affairs dataset revealed anti-TNF therapies are associated with an increased risk of pneumonia and hospitalization; though this was not stratified by age, 36.5% of persons in this study were >64 years (38). In a meta-analysis, elderly persons with IBD (≥60 years) exposed to biologics were at increased risk of both serious infections (random effects summary relative risk: 2.70; 95% CI: 1.56, 4.66; *I*^2^ = 57%) and opportunistic infections (random effects summary relative risk: 3.16; 95% CI: 1.09, 9.20; *I*^2^ = 73%), though the quality of evidence was low to very low (39).

In a pooled analysis of data from randomized trials assessing anti-TNF use, elderly people (≥60 years) with ulcerative colitis had a higher increased risk of serious adverse events, but this increase in risk could not be attributed to anti-TNF therapy (21). In a systemic review and meta-analysis of those with immune-mediated inflammatory diseases (IBD, rheumatoid arthritis, and psoriasis), those ≥60 years on biologics had an increased risk of infection compared with younger persons on biologics, and an increased risk compared with older persons not on biologics (40).

Vedolizumab has a lower risk of infection-related complications (41), and therefore considered safe for all ages (42). In a retrospective study of persons with IBD on vedolizumab, there were more infections in the elderly group (12% vs. 2%), though the authors noted the increased number of infections could be related to age or underlying disease (26). In a cohort of 497 persons with IBD >64 years, vedolizumab was as safe as 5-ASA medications with a similar risk of severe infections (38.5 vs. 30.6 events per 1000 person-years) (43). In a study of persons >60 years comparing vedolizumab with anti-TNF therapy, there was no difference in serious infections (log rank, *P* = 0.43) (27). In people >60 years, there was no difference in the risk of infection comparing participants on anti-TNF or vedolizumab (20% vs. 17%, *P* = 0.54); pneumonia was the most common infection (28). Another study included persons with IBD ≥65 years, those on vedolizumab had a lower rate of infection-related hospitalizations (aHR: 0.47; 95% CI: 0.25, 0.86), compared to those on anti-TNF therapies (44).

Malignancies were infrequent in people >60 years, with no differences seen between anti-TNF and vedolizumab when assessing skin cancers (0.8% vs. 1.0%, *P* = 0.86) and other malignancies (3% vs. 1%, *P* = 0.27) (28). In the GEMINI long-term safety study, the number of malignancies observed was as expected for the general population (45). The risk of malignancy (excluding nonmelanoma skin cancers) among people with IBD >64 years was similar among people with IBD receiving vedolizumab and 5-ASA medications (17.6 vs. 15.6 events per 1000 person-years) (43).

Ustekinumab is also considered safe in the treatment of IBD. In a retrospective study of 117 persons with Crohn’s disease on ustekinumab, there were no differences in infections (5.2% vs. 7.7%, *P* = 0.7), infusions reactions (2.6% vs. 6.4%, *P* = 0.77), or postsurgical complications (*P* = 0.99) in those >65 years compared with those <65 years (29).

### Combination Therapy (anti-TNF and Thiopurine)

In those >60 years with Crohn’s disease, there were no differences in infection risk when comparing early use of combination therapy (anti-TNF and thiopurine together) and conventional treatment (23). In a large US study using health administrative data from Veterans Affairs, the incidence of herpes zoster infection was higher in persons with IBD when compared to those without IBD (aHR: 1.72; 95% CI: 1.51, 1.96), with the highest risk being observed in those >60 years on combination thiopurine and anti-TNF therapy (11).

### JAK-Inhibitors

Tofacitinib is known to increase the risk of herpes zoster infection (46). Tofacitinib is also known to increase the risk of venous thromboembolism, especially in those with known risk factors for thromboembolism, independent of the risk posed by ulcerative colitis (one individual with deep vein thrombosis, IR: 0.04; 95% CI: 0.00, 0.23, four individuals with pulmonary embolism, IR: 0.16; 95% CI: 0.04, 0.41) (47).

## AGE-RELATED COMORBIDITIES

IBD itself is associated with an increased risk of certain comorbidities, and as persons with IBD age, they are more likely to develop these and other age-related comorbidities. In a longitudinal study, spanning 21 US state inpatient databases and accounting for almost half of the population, older individuals are more likely to have two or more comorbidities, based on the Charlson Comorbidity Index, compared with younger individuals (39% vs. 4%) (12). Individuals with IBD have an increased risk of ischemic heart disease (RR: 1.244; 95% CI: 1.142, 1.355) (48).

Persons with IBD are at increased risk of osteoporosis, and while corticosteroid use is a known risk factor as well, this risk cannot be attributed to steroid use alone (49). A population-based study from Manitoba revealed that comorbidities were increased both in pre- and post-IBD diagnosis (50). After being diagnosed with IBD in those >65 years, there was an increased risk of cardiac disease (HR: 1.24; 95% CI: 1.07, 1.43), cerebrovascular diseases (HR: 1.19; 95% CI: 1.01, 1.40), peripheral vascular disease (HR: 1.36; 95% CI: 1.14, 1.62), COPD (HR: 1.38; 95% CI: 1.12, 1.70), cancer (HR: 1.21; 95% CI: 1.04, 1.40), diabetes (HR: 1.17; 95% CI: 1.01, 1.35), among other comorbidities.

A cohort study of 1480 persons with IBD in China looked at those >60 years compared with those <60 years and found that older people had an increased incidence of cancer (26.9 vs. 9.51 per 1000-person years), with colorectal cancer being the most common (51). Persons in the elderly group had a shorter time from IBD diagnosis to developing cancer, and the presence of diabetes was a risk factor in the progression to cancer.

Polypharmacy is also an issue in the elderly, perhaps contributing to nonadherence with IBD therapies. In a retrospective study 393 persons with IBD >64 years were prescribed a mean number of 7.0 drugs (52). Frailty is more common in the elderly, and this is associated with worse outcomes in IBD, including mortality (aHR: 1.57; 95% CI: 1.34, 1.83), prolonged hospitalization (median 9 days vs. median 5 days, *P* < 0.01), and readmission for severe IBD (aHR: 1.22; 95% CI: 1.16, 1.29) (53,54). One study showed that frailty, when present pretreatment, was associated with an increased risk of infections (19% vs. 9% for anti-TNF and 17% vs. 7% for immunomodulators, *P* < 0.01 in both groups) (55). It is unknown, however, if pretreatment frailty is a predictor of IBD outcomes by drug class. More evidence is needed in this field.

Deficits in geriatric assessment are common in the elderly with IBD, which are associated with a lower health-related quality of life based on the short IBD questionnaire (mean score in those with severe deficits 50.7 vs. 58.4 in those with moderate deficits, and 62.3 without deficits, *P* < 0.001), and those with active disease are more prone to having deficits (39.9% clinically active disease in severe deficits, 27.7% in moderate deficits and 14.9% in those without deficits, *P* = 0.001) (56).

## ACCESS TO CARE

Unfortunately, there are not many studies assessing access to care in seniors. A population-based study in three provinces (Manitoba, Ontario, and Alberta) reported persons with IBD >65 years in rural households were less likely to receive care from a gastroenterologist for their IBD compared to seniors in urban areas (OR: 0.35; 95% CI: 0.26, 0.46) (57). A retrospective study assessing Veterans Health Administrations data in the US showed a higher level of continuity of care within one year was associated with being >65 years, compared with a younger cohort; this is important because a lower level of continuity of care in their study was associated with worse outcomes (58).

As virtual care becomes increasingly common in the management of IBD, it may be difficult for older persons to access their IBD care, online questionnaires, or digital applications due to a decrease in technology literacy. One study showed that the self-reported mean information technology literacy scores worsened with advancing age (59). Further information on virtual care models can be found in Mathias et al. (this volume). Utilization of gastroenterology care in those with elderly-onset IBD varies, and those treated by gastroenterologists or in networks with more gastroenterologists have better outcomes (60). In the same study, surgery in persons with Crohn’s disease was not associated with the availability of a gastroenterologist or access to specialist care, though there was variation in the five-year risk of colectomy in persons with ulcerative colitis (60). Care by a gastroenterologist was associated with immunomodulator and biologic use within five years, as compared to those whose primary provider was not a gastroenterologist (60).

## COSTS

A retrospective analysis from 2007 to 2016 of pharmacy and administrative claims data in the US revealed that elderly persons with IBD had higher costs of care when compared to persons with IBD aged 35 to 44 (61). Nguyen et al. reported that persons ≥65 years had longer hospital stays than those who were middle-aged and younger (median of stay seven days compared with six days and five days, respectively), as well as an increased mortality (12). This ultimately led to significantly higher hospitalization costs for older persons with IBD, with a mean of USD $15,078 vs. USD $12,921 and USD $10,070, respectively. Despite this, older persons with IBD were less likely to undergo gastrointestinal surgeries or IBD-related procedures. A population-based study within Manitoba revealed that mean costs directly attributable to Crohn’s disease and ulcerative colitis were lowest in those >65 years old when compared with younger cohorts (62).

## VACCINES

Elderly persons with IBD are at increased risk of opportunistic infections (63), and vaccines are an important protective mechanism. It is recommended that those over 50 receive the recombinant herpes zoster vaccine and that those over 65 receive the pneumococcal vaccine (64). Therapies to treat IBD are associated with an increased risk of infection, as anti-TNF inhibitors are associated with an increase in pneumonia, but vaccination against pneumococcus is associated with a reduced risk of pneumonia (65). While receiving the recombinant herpes zoster vaccine can help reduce one’s risk of acquiring the herpes zoster infection, persons with IBD are still at increased risk of acquiring the infection even after getting vaccinated, compared with those who do not have IBD (66). Older age (this threshold varied in studies, from >50 to >65 years) is associated with an increased uptake of influenza and pneumococcal vaccine, although they were less likely to get the live-attenuated herpes zoster vaccine (67).

Persons with immune-mediated inflammatory diseases (including IBD) aged >60 years on immunomodulators or biologics who received COVID-19 vaccines had reduced odds of a positive antibody response following vaccination with either mRNA or adenovirus-vector vaccines (68), and antibody levels decline in seniors at a faster rate than in younger populations. In persons with IBD vaccinated with mRNA or nonviral vector vaccines, age >60 years was associated with lower anti-SARS-CoV-2 antibodies and lack of antibody response (69,70). More on overall vaccine response to COVID-19 vaccines can be found in Kaplan et al. (this volume).

## CONCLUSION

The prevalence of IBD in seniors is increasing, and this provides many different challenges due to the presence of comorbidities, frailty, polypharmacy, and an increased risk of infections and malignancies. While treatment approaches are similar to nonelderly persons with IBD, the use of biologics in real-world cohorts is lower in elderly persons with IBD. Whether anti-TNF is associated with an increased risk of infections is controversial, and newer biologics including vedolizumab and ustekinumab are thought to have a more favorable safety profile when treating elderly persons with IBD. Access to care for seniors can be difficult, as technology advances with online questionnaires and digital applications on smartphones, though outcomes are better when seniors’ IBD is treated by a gastroenterologist. Vaccines are an important mechanism to prevent infections and should be offered to all elderly persons with IBD.

## KNOWLEDGE GAPS AND FUTURE RESEARCH DIRECTIONS

1 It is important to better define the natural history of IBD in the elderly, and how it differs from other age groups with IBD.
2 A better understanding of how to optimize biologic therapies when treating elderly persons with IBD is needed.
3 The magnitude of infection risk with older advanced therapies (e.g., anti-TNF) and newer advanced therapies (e.g., anti-IL-23, integrin inhibitors) needs to be defined for the elderly, including those with multiple comorbidities. Randomized trials should include a larger number of elderly persons with IBD so that efficacy, and safety, can be better defined.
4 Further studies are needed on how, and why, costs are different in the elderly population with IBD, and regarding cost-effective measures in lowering both their direct and indirect costs.
5 An understanding of the spectrum of technology literacy among seniors with IBD is important as we enter an era of increased virtual care and remote monitoring of IBD status.
6 The financial, social, and time costs for family members of elderly with IBD is not defined, but, in some cases, can rival or exceed that for family members of children with IBD. This can be especially problematic for family members of the elderly persons with mobility and cognitive issues.
7 Research into optimizing multidisciplinary care for seniors living with IBD, including communication and shared decision making among different medical specialists, healthcare providers and social services, is required.

## PATIENT AND CAREGIVER PARTNER PERSPECTIVE

Patient partners acknowledged that the number of seniors with IBD is increasing due to new diagnoses, partly because of new diagnoses in older adults, and partly because adults who already have IBD are aging. Patient partners recognize that the management of IBD in seniors is complicated due to the presence of underlying conditions, polypharmacy, frailty, and lack of access in some areas to specialized care/gastroenterologists. Some medications may be safer to use and offer fewer adverse effects than others in the senior population. Encouraging vaccinations in seniors with IBD is important in protecting against infections. Seniors with IBD want to be engaged in their own care and encouraging greater access to care can assist with that engagement. Virtual technology can be used as one method to enhance access; however, support may be needed for its use and connecting virtually may not be preferred by all. Another means to enhance care assess is to offer greater in-home care services for seniors. IBD in seniors is a growing public health issue requiring greater research and coordinated, interprofessional resources to address complex care needs to allow seniors to make informed decisions about their care. Partners stressed the importance of self-advocacy or having someone to advocate on their behalf for their care needs.

## POLICY IMPLICATIONS AND KEY ADVOCACY OUTCOMES

1 Ensuring that seniors with IBD have access to gastroenterology care is essential; remote access is also important because there may be barriers in some forms of virtual care. Telemedicine should be accessible and be funded to ensure it can be utilized by those who need it (e.g., those with mobility restrictions or living in remote locations).
2 There is a need for services to allow seniors, or someone advocating for the senior, to review their medications with a pharmacist as needed.
3 A social worker or support system for peer advocates/support is needed to ensure seniors have sufficient resources to purchase their medications and food and to be a healthcare advocate for those who cannot provide/advocate for themselves.
4 Senior-specific support groups would be useful to help individuals not feel isolated.
5 Support should be provided for house-bound—physically or by choice—individuals by increasing access to in-home care (where possible).

## SUPPLEMENT SPONSORSHIP

This article appears as part of the supplement “The Impact of Inflammatory Bowel Disease in Canada in 2023”, sponsored by Crohn’s and Colitis Canada, and supported by Canadian Institutes of Health Research Project Scheme Operating Grant (Reference number PJT-162393).

## Data Availability

No new data were generated or analyzed in support of this review.
